# *Bacillus aryabhattai* TFG5-mediated synthesis of humic substances from coir pith wastes

**DOI:** 10.1186/s12934-021-01538-x

**Published:** 2021-02-17

**Authors:** Iniyakumar Muniraj, Syed Shameer, Priyadharshini Ramachandran, Sivakumar Uthandi

**Affiliations:** Biocatalysts Laboratory, Department of Agricultural Microbiology, Tamil Nadu Agricultural University, Coimbatore, Tamil Nadu 641 003 India

**Keywords:** *Bacillus aryabhattai* TFG5, Tyrosinase, Laccase, Coir pith biomass, Oxidative polymerization, Humic substances synthesis

## Abstract

**Background:**

Humic substances (HS) form the largest proportion among all the constituents of soil organic matter and are a key component of the terrestrial ecosystem. HS plays a multifunctional role in the environment by controlling the biogeochemical carbon cycle, providing nutrients and bio-stimulants for plant growth, and interacting with inorganic and organic pollutants. The rate of formation of HS in soils determines its productivity and carbon sequestration capacity. Enhancement of HS synthesis in the soil through the microbial route not only increases CO_2_ sequestration but also mitigates the greenhouse gas emissions in the environment.

**Result:**

In this study, we attempted to understand the mechanism of formation and enhancement of HS from coir pith wastes using the tyrosinase produced by *Bacillus aryabhattai* TFG5. The bacterium TFG5 isolated from the termite garden produced the tyrosinase (1.34 U mL^−1^) and laccase (2.1 U mL^−1^) at 48 h and 60 h of fermentation, respectively. The extracellular tyrosinase from *B. aryabhattai* TFG5 was designated as TyrB. Homology modeling of TyrB revealed a structure with a predicted molecular mass of 35.23 kDa and two copper ions in the active center with its conserved residues required for the tyrosinase activity. TyrB efficiently transformed and polymerized standard phenols, such as *p*-cresol, *p*-hydroxyl benzoic acid, Levo DOPA, and 2,6 DMP, besides transforming free phenols in coir pith wash water (CWW). Additionally, UV–Vis and FT-IR spectra of the degradation products of the coir pith treated with TyrB revealed the formation of HS within 3 days of incubation. Furthermore, the E472/664 ratio of the degradation products revealed a higher degree of condensation of the aromatic carbons and the presence of more aliphatic structures in the HS.

**Conclusion:**

The results confirmed the influence of TyrB for the effective synthesis of HS from coir pith wastes. The results of the present study also confirm the recently accepted theory of humification proposed by the International Humic Substances Society.

## Background

Soil fertility and productivity are central to meet the global demand and consumption of crops for food and feed requirements. Soil productivity is determined by soil organic matter. Humic substances (HS) form the largest proportion among all the constituents of soil organic matter. Enhancing the rate of formation of HS is essential to increase soil productivity as well as to model the soil carbon flow [1]. In recent years, soil carbon flow modeling has played a vital role in predicting future carbon emission patterns in soil, assisted in the development of mitigation strategies to restrict soil CO_2_ emission, and offered ways to increase soil carbon sequestration for improved soil productivity [2]. One of the most promising ways to reduce CO_2_ emission from the soil and enhance soil productivity is to improve the HS synthesis in soil [3]. HS are formed by the oxidative polymerization of phenols in soil. Phenols are small aromatic molecules produced by the depolymerization of lignin in plant litter. Soil enzymes, Fe(III)–Fe(II), Mn(IV)–Mn(III)–Mn(II) catalysts, and soil microorganisms play a significant role in the formation of HS in soil. Oxidoreductases, including tyrosinases and fungal laccases, are enzymes capable of polymerizing small phenolic compounds released during the depolymerization of lignin. Such polymerization of phenols enables them to cross-link with amino acids, sugars, and other aromatic molecules. Then, a supramolecular structure of HS is formed. HS are bound by hydrophobic and ionic bonds and serve as a stable part of the soil organic matter [4, 5].

HS assist in increasing the soil organic matter, increasing the nutrient and water holding capacity of the soil, and improving soil health by binding to soil pollutants. The resident time of HS in the soil is 10^2^–10^3^ years, and thus, the HS formation in soil (humification) is considered one of the critical processes in the atmospheric carbon dioxide sink. The global soil carbon (C) pool is estimated to be 2500 gigatons (Gt), which includes about 1550 Gt of soil organic carbon (SOC) and 950 Gt of soil inorganic carbon (SIC). The soil C pool is 3.3 times the size of the atmospheric pool (760 Gt) and 4.5 times the size of the biotic pool (560 Gt) [6]. Therefore, increasing the rate of HS formation is vital for the long-term storage of carbon in the soil, besides its indirect role in reducing global warming.

Since HS form the largest pool of recalcitrant organic carbon in the soil, their formation, oxidative biotransformation, and mineralization are predominantly due to the presence of oxidative enzymes, mainly of fungal origin [7]. The peroxidases, mainly lignin peroxidase (LiP), Mn-dependent peroxidase (MnP), versatile peroxidase (VP), laccase, and tyrosinase, are the major oxidative enzymes involved in the formation of HS [8–11]. Among these enzymes, tyrosinase catalyzes the oxidation of the phenolic and non-phenolic portions of the substrate into quinones and aryl radicals, respectively. HS formation catalyzed by fungal tyrosinase in wood and soil has been reported previously [9]. Bacterial tyrosinases with industrial applications are also reported. For example, solvent-tolerant tyrosinase for enhanced activity under solvents and tyrosinases for water decontaminantion, phenol removal from wastewater, and detoxification of plant host defenses have been reported [10, 12]. Nevertheless, the literature related to bacterial tyrosinase and its involvement in HS formation is scarce. It is essential to elucidate the mechanisms of HS formation catalyzed by bacterial tyrosinase. Hence, in this study, we aimed to understand the role of bacterial isolate *Bacillus aryabhattai* TFG5 in tyrosinase production and its involvement in HS production using coir pith wastes. In addition, the bacterial enzyme was evaluated for the oxidation of phenols from coir pith wash water (CWW).

## Results

### Time course of tyrosinase and laccase production

**Fig. 1:**
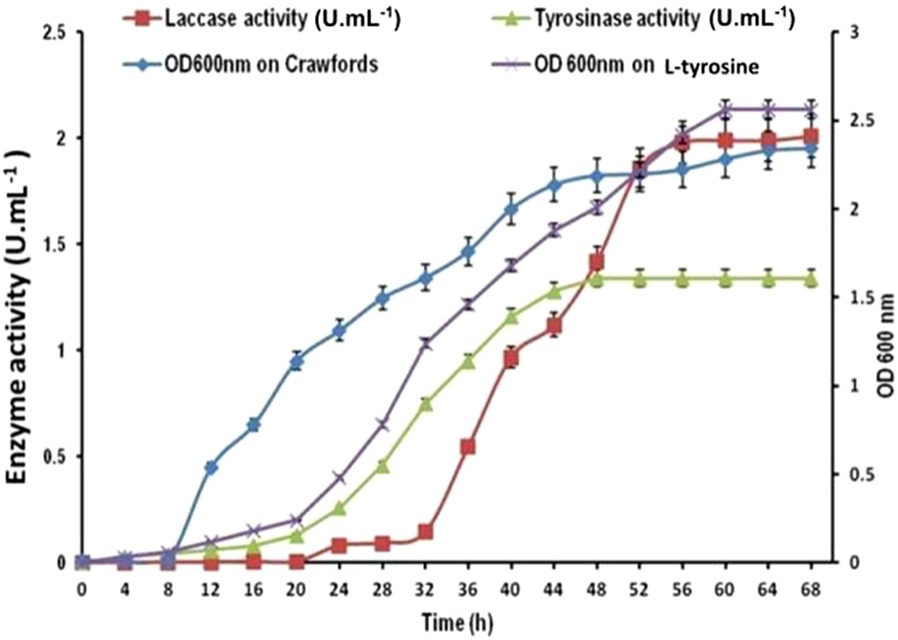
Time course production of tyrosinase and laccase by *B. aryabhattai* TFG5. Enzyme activities were estimated at 4-h intervals in the respective media, and the optical density of 600 nm measured for the growth of *B. aryabhattai* TFG5. Values are the means of three replicates, and the error bar indicates the standard deviation

In this study, a potential tyrosinase and laccase-producing *B. aryabhattai* TFG5 was evaluated for HS formation. According to the time course studies, the tyrosinase secretion by TFG5 started at 20 h, then gradually increased, and finally reached its maximum value at 48 h (activity 1.34 U mL^−1^), while the growth of the bacterium in tyrosine broth started 4 h later and reached the maximum value at 60 h. Similarly, laccase activity (0.2 U mL^−1^) began after 20 h of fermentation, and the maximum value was reached at 68 h (activity 2.1 U mL^−1^), while the highest bacterial growth was also observed at 68 h of fermentation in Crawford’s broth (Fig. 1).

### Bioinformatics analyses

**Fig. 2:**
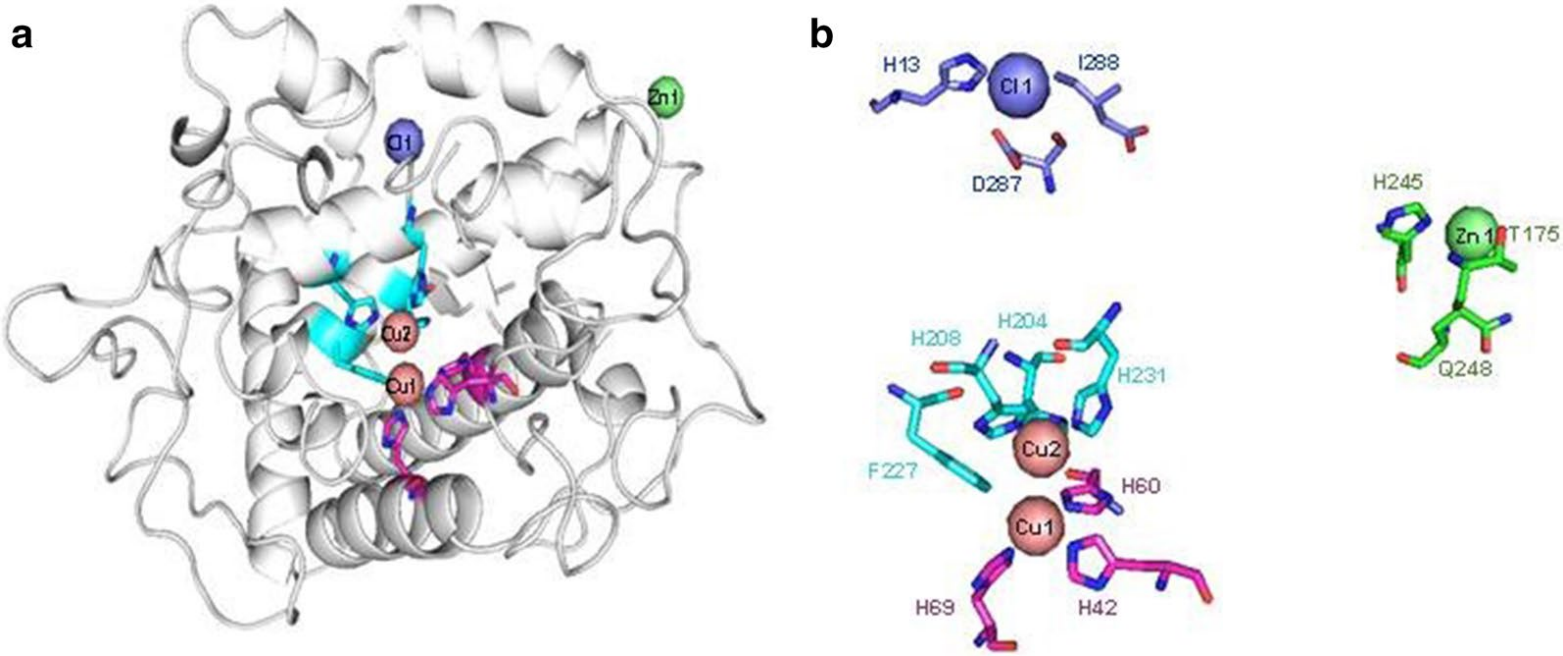
Homology model of the TryB of *B. aryabhattai* TFG5. TyrB was predicted using the crystal structure of *B. megaterium* (3NM8) (**a**). There were three Cu ligands in the model (each indicated by a sphere), each of which had contact with His residues and the Phe residue (**b**)

Gene-specific primers of *B. megaterium* were used for the amplification of the tyrosinase gene in *B. aryabhattai* TFG5 and that of the ORF encoding the gene (MW505907). The sequence had 100% identity with *B. megaterium* (CP010586) and *B. aryabhattai* (MT271685). The homology model of TFG5 was translated into protein and the protein structure was predicted in the SWISS homology modeling server using *B. megaterium* tyrosinase (PDB: 3NM8) as a template. The structure and the corresponding metal-binding domain are shown in Fig. 2. The translated protein had a predicted molecular mass of 35.23 kDa and was found to be a bi-copper protein in which Cu1 coordinated with three histidine residues (H69, H42, H60) and Cu2 coordinated with three histidines (H204, H208, H231) and one phenylalanine (F227) residue. Other than copper, the protein also contained Cl and Zn binding sites.

### Oxidative polymerization of phenolic compounds

**Fig. 3:**
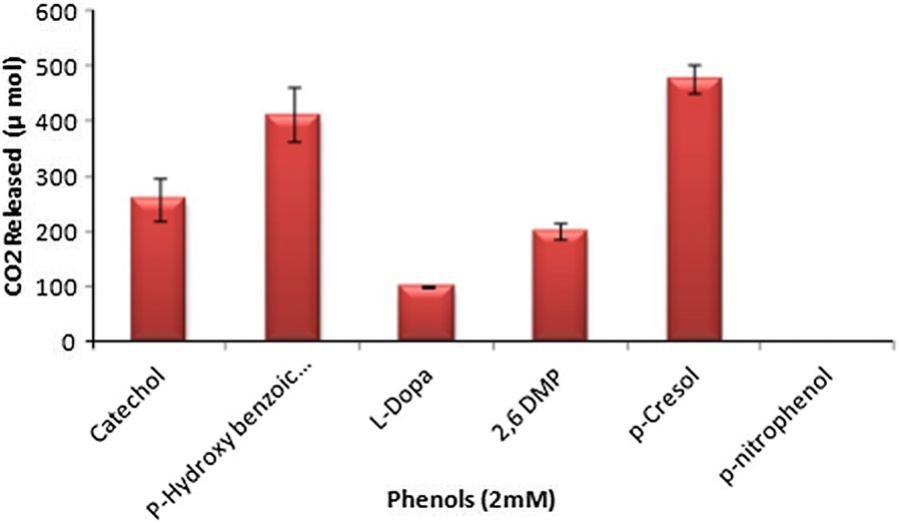
Oxidative polymerization of phenols by the extracellular enzyme of *B. aryabhattai* TFG5. Mono and diphenols were evaluated at 2 mM concentration for oxidative polymerization, and their respective CO_2_ release was monitored

TyrB influences monophenol depolymerization rather than diphenol depolymerization, as evidenced by the increase in the release of CO_2_ to the former rather than the latter. The maximum CO_2_ release was observed in *p*-cresol (477.25 µmol) followed by *p*-hydroxybenzoic acid (412.5 µmol), while the lowest CO_2_ release was observed in l-DOPA followed by 2,6 DMP (Fig. 3). Although the enzyme could oxidatively polymerize mono- and di-phenols, *p*-nitrophenol was not oxidized by this enzyme. The reason for this is unclear and requires further investigation. Furthermore, the FT-IR spectra of the transformed products revealed additional functional groups, suggesting the formation of reaction products and confirming the ring cleavage of the phenols. There were no differences between the functional groups of *p*-nitrophenol and 2,6-dimethoxy phenol when treated with the enzyme. On the other hand, the presence of two additional functional groups at wavenumbers 1655.59 cm^−1^ and 2185.92 cm^−1^ were observed in the absorption spectrum of *p*-cresol, which corresponded to alkenes C=C medium stretching and alkynes CΞC stretching, respectively. Similarly, the presence of an additional functional group of alkynes CΞC stretch was noticed at 2147.35 cm^−1^ for l-DOPA and catechol. Strong aromatic amines at wavenumber 1327.5 cm^−1^ were noticed for *p*-hydroxybenzoic acid treated with the enzyme from *B. aryabhattai* TFG5 (Additional file 1: Table S1).

### Transformation of CWW and humification of coir pith biomass

**Fig. 4:**
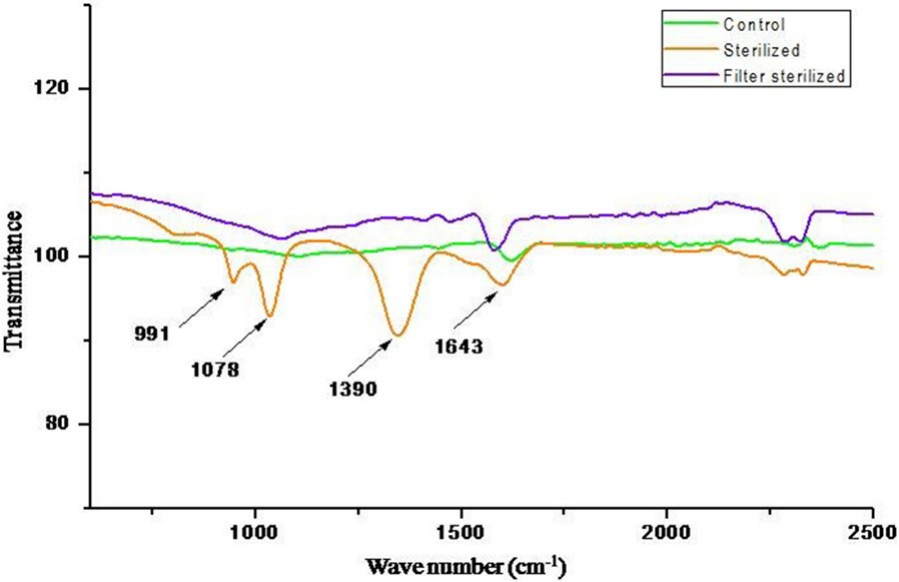
FT-IR spectra of phenols from the CWW treated with *B. aryabhattai* TFG5. FTIR peaks of the transformed products of control-sterilized and filter-sterilized CWW are presented. The presence of additional peaks for sterilized phenols is indicated by arrows

The transformed products obtained from CWW are presented in Additional file 1: Table S2. The results revealed that the number of functional groups after incubation with TyrB was higher in sterilized CWW than that in the filter-sterilized and untreated CWW. The FT-IR spectra of transformed compounds due to TyrB of CWW are presented in Fig. 4. The spectra show the presence of more functional groups in higher intensities for sterilized CWW compared to the control and the filter-sterilized CWW.

### Production of humic polymer using coir pith as a substrate

**Fig. 5:**
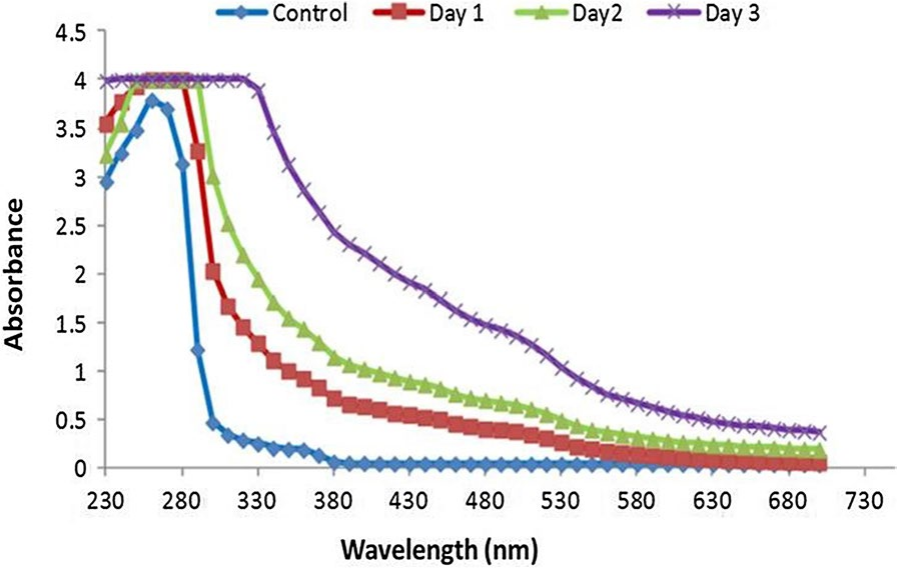
Absorbance spectra of the liquid portion of coir pith biomass after treatment with *B. aryabhattai* TFG5. Day-wise spectral scan of the treated sterilized CWW showing a hump above 480–530 nm, which is related to humic substances

**Fig. 6:**
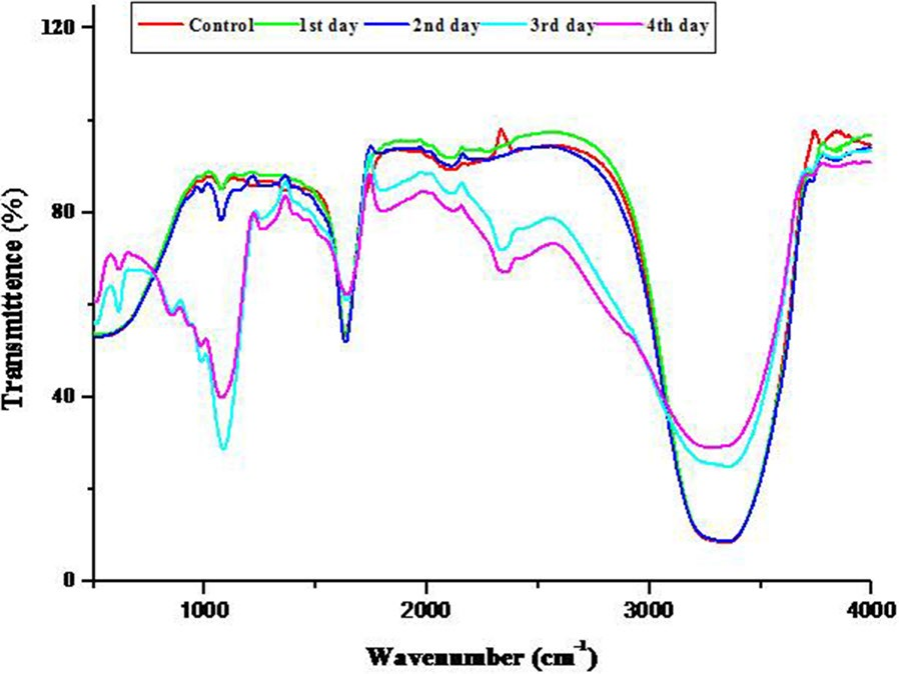
FTIR spectra of the functional groups of coir pith biomass. The spectral data were recorded at the rate of 64 scans/s from 400 to 4000 cm^−1^. The intensities of transmittance showed increased concentrations of the products released

The UV–Vis and FT-IR spectra suggested that TyrB could produce HS from the coir pith biomass within three days, as evidenced by an increase in the absorbance compared to control. The maximum increment was observed on day 3. The most dramatic increment in the absorbance was recorded from 380 to 530 nm (Fig. 5). The HS formed were characterized using several absorbance coefficient ratios. The E280/472 ratio describes the presence of aromatic groups in the HS. The coefficient increments on day 2 were decreased on day 3, showing the depolymerization of tyrosinase. Similarly, the coefficient E472/664 is associated with condensation of the aromatic carbon chains. Further results on the functional groups associated with HS (FT-IR) are shown in Fig. 6.

The FT-IR spectral data showed the presence of an aldehyde group (R–CH–O) at wavenumber 860 cm^−1^, which was not observed on the first 2 days and was noticed at high intensity on day 3; the intensity subsequently reduced on day 4. Similarly, the increased intensities of -OH groups were recorded as time progressed, as evidenced by the changes in the vibrations at wavenumber 1053 cm^−1^. The presence of alkenes at wavenumber 1641 cm^−1^ indicates the formation of the backbone of HS (Fig. 6).

## Discussion

Synthesis of HS in the soil is an oxidative process aided by the extracellular secretion of oxidative enzymes, namely tyrosinase and laccase, by wood-degrading and soil-inhabiting microbes. Tyrosinase production is reported from several fungi [13]. However, studies focused on the optimization of the process parameters for bacterial tyrosinase production are limited. Sambasiva Rao [14] optimized the conditions of tyrosinase production from *Streptomyces antibiotics* and reported a maximum activity of 4.62 U mL^−1^ [14]. In another study, recombinant *Escherichia coli* produced tyrosinase at a high level of 13 U mL^−1^ [15]. Comparatively, in the present study, *B. aryabhattai* TFG5 produced 1.34 U mL^−1^ of tyrosinase and 2.1 U mL^−1^ of laccase, and no optimization studies were performed, suggesting that this bacterium would be advantageous and a suitable candidate for HS formation in soil due to its unique property of producing both laccase and tyrosinases. In order to predict the molecular weight of the produced tyrosinase and understand its structural functions, the tyrosinase gene from TFG5 was amplified and sequenced. The gene sequence had 100% identity with the tyrosinases from *B. megaterium* and *B. aryabhattai*. The protein modeling of *B. aryabhattai* TFG5 tyrosinase with the crystal structure of *B. megaterium* tyrosinase (PDB: 3NM8) as a template was attempted. The sequence and structure analysis through multiple alignments of tyrosinase amino acid residues of *B. aryabhattai* TFG5 protein sequences revealed the conservation of the Cu1 and Cu2 binding residues with the other tyrosinases reported previously [16] (Additional file 1: Fig. S1). The results suggested that TyrB is a bi-copper protein similar to other tyrosinases.

One of the critical reactions in humification is the oxidative polymerization of phenolic compounds by tyrosinases, which subsequently yields HS in the environment [4]. The ability of TyrB to generate CO_2_ through the oxidative polymerization of phenols is directly proportional to the cleavage of the phenol rings. Such a ring-opening of phenols enables rapid degradation, which is otherwise non-reactive [17, 18]. Moreover, the results of the ATR-FT-IR showed the addition of the HS backbone in the tested phenols. It is believed that alkenes, alkynes, and aromatic amines react with the other amino acids present in the environment to form HS [19–22].

In order to elucidate the other functions, besides HS synthesis of TyrB, the phenol-rich CWW was transformed using TyrB to reduce its phenolic content. The FT-IR spectra of the transformed products exhibited additional functional groups at the wavenumbers 991.232 cm^−1^, 1078.98 cm^−1^, 1390.42 cm^−1^, and 2085.64 cm^−1^ at higher intensities, which indicated the efficient transformation of CWW by the enzyme (Additional file 1: Table S2). On the contrary, the spectra of filter-sterilized CWW did not have such additional functional groups (Fig. 4). This indicates the ability of TyrB in oxidative transformation followed by polymerization ability. While comparing the different products formed during the incubation, it was noticed that untreated control had only an aromatic ring structure, which indicated the presence of the phenolic group of compounds. Similar functional groups with modification were also observed in the sterilized phenols, indicating ring cleavage and polymerization due to enzyme [23, 24]. In an aqueous environment, tyrosinase oxidizes low molecular weight phenols and mediates polymerization, thereby precipitating the phenols. This enables easy removal of the phenolic compounds from wastewater [25]. The study reveals the presence of tyrosinase-polymerized phenols in the FT-IR spectra.

Production of HS from coir pith biomass was confirmed by the sudden shift in the absorbance at 480 nm (associated with HS) in the UV–Vis spectrum. Similarly, the increment in the absorbance over three days was attributed to the compounds with carboxylic and phenolic groups. Similar patterns of absorbance and molecules were observed when cotton stalk biochar was depolymerized using fungal oxidoreductase [22]. Furthermore, pure humus, when subjected to a sequential chemical extraction process and analyzed using advanced analytical techniques, signified the concept of humification (humeomics). In the humeomic approach, the largest fractions of humus consisted of carboxyls and phenols. The degradative products of coir pith in the present study also had compounds similar to those observed in the humeomic approach, i.e., phenolics and carboxyls [5]. Moreover, the maximum absorbance of coir pith wash water (CWW) at 280 nm and 340 nm was related to the presence of aromatic compounds, probably due to the depolymerization of the phenols of CWW. Similarly, higher absorbance at 360 nm until 472 nm indicated the presence of abundant aromatic compounds such as HS. Decreasing absorbance at 436 nm over the three days indicated the presence of molecules with more aliphatic, carbohydrates, and nitrile compounds (Table 1). Higher values of this coefficient indicated the presence of more aliphatic structures and less aromatic structures. Several studies on pure soil humus have reported a major portion of aliphatic structures [21, 26–28].

**Table 1:** Relationship between absorbance ratios, the formation of humic substances and other products in the liquid phase of coir pith biomass

Absorbance ratios	Day 1	Day 2	Day 3
E270/400	1.00	1.00	0.98
E465/665	3.99	4.34	4.31
E250/365	1.00	1.00	0.98
E280/472	1.18	1.30	1.23
E280/664	4.51	5.36	5.02
E472/664	3.81	4.12	4.09

HS, the word itself, has gained controversy over the past few years. The term ‘soil organic carbon’ was conveniently used interchangeably with humus for several years. However, the recent debate on HS suggests that HS and non-HS are not parts of humus. Scientists believe that the concept of HS should be set aside as it did not contain the compounds of alkali-extracted soil organic carbon [29, 30]. Moreover, the traditional humification pathway was questioned, and a new continuum model of soil organic matter was established, in which the formation of soil organic matter is a result of the mineralization of preferential organic molecules rather than the progressive decomposition of the plant and animal macromolecules [29].

However, increasing evidence in support of the traditional view of humic substance synthesis [3, 4] has also been reported. The traditional concept of the humification hypothesis is that the soil added plant residues undergo decomposition by the extracellular enzyme secreted by soil microorganisms. The macromolecules such as starch, protein, lipids, carbohydrates including cellulose and hemicellulose, and lignin undergo enzymatic breakdown, and the respective degradation products such as simpler sugars, amino acids, fatty acids, and phenols are released. The phenols are polymerized by several phenol oxidases, namely, tyrosinases and laccases, which give rise to phenol radicals that help in polymerization. During the polymerization, new products are formed, which are termed as humic substances (HS). The present study explored the role of bacterial tyrosinase in the formation of HS from coir pith wastes. In the present study, UV–Vis and FT-IR spectra of the coir pith biomass after treatment with TyrB showed the presence of aldehydes and acids initially at higher intensities on day 3 (Fig. 6). This signifies the products of lignin breakdown by the enzyme. Further, the absence of aldehydes and acids on day 4 suggested that these might have undergone polymerization, as evidenced by an increase in the absorbance (Fig. 5 and Table 2). The enzymatic fractionation of the biomass undergoing humification was also rich in aryl and alkyl carbon. Similar results were observed in the chemical fractionation of soil humus [5]. Therefore, the results of the present investigation are in line with the concepts and views propounded by traditional humification [3, 4]. More particularly, TyrB enhances HS synthesis in coir pith biomass. However, a more detailed humeomic approach involving the enzymatic fractionation of humus is warranted for more insights.

**Table 2:** UV–Vis absorbance of the liquid phase of coir pith biomass treated with extracellular enzyme of *B. aryabhattai* TFG5

Absorbance analyzed (nm)	Day 1	Day 2	Day 3	Day 4
280	4.00 ± 0.0	4.00 ± 0.0	3.90 ± 0.01	3.50 ± 0.01
340	4.00 ± 0.0	4.00 ± 0.0	4.00 ± 0.0	4.00 ± 0.0
360	4.00 ± 0.0	4.00 ± 0.0	4.00 ± 0.0	4.00 ± 0.0
400	4.00 ± 0.0	4.00 ± 0.0	4.00 ± 0.0	4.00 ± 0.0
436	3.81 ± 0.01	3.57 ± 0.01	3.68 ± 0.01	2.91 ± 0.02
465	3.52 ± 0.01	3.23 ± 0.01	3.35 ± 0.01	2.55 ± 0.02
472	3.37 ± 0.01	3.07 ± 0.01	3.19 ± 0.01	2.39 ± 0.02
600	1.30 ± 0.02	1.13 ± 0.02	1.17 ± 0.02	0.65 ± 0.02
664	0.88 ± 0.02	0.74 ± 0.03	0.77 ± 0.03	0.36 ± 0.03

## Conclusion

It is concluded that tyrosinase from *B. aryabhattai* TFG5 improves the synthesis of HS from coir pith biomass. Such enhanced catalytic biotransformation and formation of HS from coir pith biomass by TyrB suggest that TyrB-derived products coordinate with the chemical fraction of humus. Besides, the oxidative transformation of CWW into monophenolic units by this enzyme would find application in the detoxification and precipitation of toxic phenols in the coir industry. The HS produced through this process could be a slow-release organic fertilizer in organic agriculture, and a soil conditioner/binder in degraded soil that can enhance the carbon sequestration potential of the soil. A detailed humeomic approach would provide more insights into the HS synthesis by bacterial enzymes.

## Materials and methods

### Culture, materials, chemicals, and media

*Bacillus aryabhattai* TFG5 (GenBank accession number: KT956906) isolated from the fungal garden of termite mound was used in the present study. The bacterium secreted both laccase and tyrosinase as inducible enzymes when supplemented with respective media (Crawford’s media for laccase and l-tyrosine media for tyrosinase). The extracellular cell-free crude enzyme secreted by *B. aryabhattai* (hereafter referred to as TyrB) induced with l-tyrosine was used for all studies related to HS synthesis. Coir pith biomass and CWW obtained from local industries were used for the oxidative transformation studies. The chemicals 3-methyl-2-benzothiazolinone hydrazine (MBTH), l-tyrosine, l-DOPA, ABTS (2,2′-azino-bis(3-ethylbenzothiazoline-6-sulfonic acid), and mushroom tyrosinase were obtained from Sigma Aldrich (St. Louis, Missouri, USA). *p*-hydroxybenzoic acid, 2,6-dimethoxy phenol, *p*-cresol, *p*-nitrophenol, and catechol were obtained from HI-Media Laboratories Pvt. Ltd (Mumbai, India). Laccase production was monitored in Crawford's media containing (g/L) glucose (1.0), yeast extract (1.5), Na_2_HPO_4_ (4.5), KH_2_PO_4_(1.0), MgSO_4_ × 7 H_2_O (0.12), NaCl (0.2), and CaCl_2_(0.05). Similarly, tyrosinase production was carried out in the medium containing (g/L) casein broth hydrolysate (10), K_2_HPO_4_ (0.5), MgSO_4_ (0.25), and l-tyrosine (1). *B*. *aryabhattai* TFG5 was maintained in Luria Bertani (LB) broth containing (g/L) tryptone (10), yeast extract (5), and NaCl (10).

### Time course production of tyrosinase and laccase

One-day-old culture of *B. aryabhattai* TFG5 grown in LB broth and having an OD value of 0.1 was inoculated into 250 mL Erlenmeyer flasks containing 50 mL Crawford’s medium and l-tyrosine medium for monitoring laccase and tyrosinase, respectively. The cultures were incubated in an orbital shaker (New Brunswick, USA) maintained at 150 rpm and 30 °C. Enzyme activities were monitored at 4-h intervals for 68 h by measuring the changes in absorbance under standard assay conditions. Similarly, growth was measured by recording optical density at 600 nm in a multi-mode spectrophotometer Spectra Max 360 (Molecular Devices, USA). At regular intervals, 1 mL of an aliquot from the 250 mL flasks was aseptically transferred into a 2-mL centrifuge tube, and the contents were centrifuged to 10,000 rpm for 10 min at 4 °C in a refrigerated centrifuge (Accuspin, Thermo Fisher, India). Cell-free culture supernatants were used for determining the enzyme activity, oxidative polymerization, and humification.

### Enzyme assay

Laccase activity was determined using 1 mM ABTS at 30 °C for 5 min and then measuring the change in the absorbance at 420 nm (*ε*_max_ = 3.6 × 10^4^ M^−1^ cm^−1^) in a multi-mode microplate reader (Spectramax 360; Molecular Devices, USA). The reaction mixture contained an appropriately diluted supernatant mixed with 1 mM ABTS in sodium phosphate buffer (50 mM, pH 4.5) [31]. Tyrosinase activity was determined at 30 °C for 5 min using 1.5 mM l-DOPA (ε 505 = 2.9 × 10^4^ M^−1^ cm^−1^) [32]. The reaction mixture contained phosphate buffer (50 mM, pH 7), 1.5 mM l-DOPA, 5 mM MBTH (3-methyl-2-benzothiazolinone hydrazone), 2% N–N′-dimethylformamide, 0.1 mM sodium azide, and 10 µL of appropriately diluted supernatant. The reaction was stopped by adding 100 µL of 1 M perchloric acid, and the absorbance was measured at 505 nm. One unit of enzyme activity was defined as the amount of enzyme required to oxidize 1 µM min^−1^ of the substrate under standard assay conditions.

### Bioinformatics analyses

Tyrosinase gene from *B. aryabhattai* TFG5 was amplified using the gene-specific primers of *B. megaterium* tyrosinase (Forward 5′-GAGGTTAAACCATGGTAACAAGTATAGAG TTAGAAAAAACG-3′ and Reverse 5′-TGCTGTTTCTAGATCTGGTTAATGGTGGTGATGGTGATGTGAGGAACGTTTTGATTTTC-3′) [33]. The PCR assay was performed in a 25 µL reaction mixture consisting of 2 µL of 50 ng DNA, PCR buffer (containing MgCl_2_), 0.2 mM (forward and reverse primers), 0.2 mM (each) dNTPs, and 1 unit of Taq polymerase. The amplification was performed in Bio-Rad T100 PCR thermocycler (Hercules, California, United States), with initial denaturation at 95 °C for 4 min, followed by 35 cycles each of denaturation at 94 °C for 30 s, annealing at 58 °C for 1 min, and extension at 72 °C for 1 min, and the final extension at 72 °C for 5 min. The amplified product was purified and sequenced at Sci-Genome (Cochin, India). The gene sequences were translated into proteins, which were then aligned using the multiple sequence alignment (MSA) tool in Bio edit version 7.2.5. The protein sequences of various tyrosinases were retrieved from NCBI (www.ncbi.nlm.nih.gov) or RCSB (www.rcsb.org) data repositories, and the homology model of tyrosinase from *B*. *aryabhattai* TFG5 (TyrB) was constructed using an automated Swiss-modeling server and verified using the Structure Analysis and Verification Server version 4 (http://services.mbi.ucla.edu/SAVES/) [34]. The homology model of TyrB was visualized using open-source PyMOL version 0.97 (2004). The theoretical molecular weight of TyrB was predicted online using the compute Mw tool (http://web.expasy.org).

### Oxidative polymerization of phenolic compounds

Six different phenols, including mono (*p*-hydroxybenzoic acid; 2,6-dimethoxyphenol; *p*-cresol; *p*-nitrophenol) and di-phenols (catechol; l-DOPA), at concentration 2 mM, were evaluated for oxidative polymerization. The phenolic compounds (2 mM) were placed in 250 mL conical flasks containing 50 mL of the reaction mixture in 50 mM sodium phosphate buffer (pH 7.0). TyrB at 10.0 U mL^−1^ was added, followed by incubation in the dark for 48 h. One mL of aliquot was transferred into 2.5 mL microfuge tubes and centrifuged for 10 min at 2500 rpm in a microfuge (Bio-Rad, Hercules, USA). The oxidative polymerization of phenols was measured by following previously described methods [23, 24]. Further, 10 µL of the liquid sample was applied to the diamond Attenuated Totally Reflection (ATR) crystal and analyzed in JASCO 7000 Fourier Transform Infrared (FT-IR). The samples were subjected to IR radiation to obtain the corresponding spectra, and the average was computed from several data acquisitions. The FT-IR spectra were generated in the wavenumber range of 700–4000 cm^−1^ with a resolution of 4 cm^−1^. After each measurement, the crystalline surface was washed with acetone and dried using a soft paper [23].

### Transformation of coir pith wash water (CWW)

The transformation was performed using 50 mL of CWW in 250 mL Erlenmeyer flasks under both sterile and non-sterile conditions. Filter sterilization of CWW was carried out by passing the CWW through 0.24-µ syringe filters (Pall, Bengaluru, India). Sterilization of CWW was achieved in an autoclave (121 °C, 15 lbs for 15 min), while non- sterilized CWW was used as the control. TyrB at a concentration of 10 U mL^−1^ was added separately to a total volume of 50 mL in all the treatments, and the flasks were incubated under shaking conditions at 30 °C for 48 h. The transformation products were analyzed using an ATR-FT-IR. Infra-red spectral data were collected in the range of 4000 to 700 cm^−1^ at a resolution of 4 cm^−1^ [23, 24]. Besides, the maximum absorbance of CWW was evaluated using a multi-mode microplate reader (Spectra max 360; Molecular Devices, USA) [35], and the maximum absorbance at 300 nm was used for the ‘change in absorbance’ studies.

### Humification of coir pith biomass

Coir pith biomass was used as a substrate for the synthesis of HS using TyrB [22]. Sieved and dried coir pith biomass at 5% consistency was incubated in the presence of extracellular enzymes from *B. aryabhattai* TFG5 at 10 U mL^−1^ in 50 mM phosphate buffer at pH 7.0 for 1 week for the formation of HS [1, 19, 20]. In order to separate the solids from the liquid, the resultant samples were centrifuged for 10 min at 2500 rpm (BioRad). The liquid phase was analyzed immediately and then frozen. In contrast, the solid phase was dried at 105 °C for 24 h and then maintained at room temperature until use. The depolymerization degree and the chemical properties of the compounds in the liquid phase were determined by measuring the increment in the absorbance at 450 nm in a multi-mode spectrophotometer.

In order to evaluate the associated molecules of HS synthesis in the liquid phase, several coefficient values, such as E270/400, E465/665, E250/365, E280/472, E280/664, and E472/664, were calculated [22]. The treated and untreated solid samples were analyzed using FT-IR (JASCO 7000 Fourier Transform Infra-Red) spectroscopy. The spectral data were collected in the range of 4000 to 700 cm^−1^ with a resolution of 4 cm^−1^.

### Statistical methods

All the experiments were conducted in triplicates and the results were expressed as the mean value with standard deviation. The results were analyzed by Duncan’s test and one way ANOVA for statistical analysis. The *P*-value was calculated and the significance threshold was established at 0.005. All statistical analyses were performed in the MATLAB software.

## Supplementary Information

**Additional file 1: Figure. S1.** Multiple sequence alignment of TyrB: MSA was performed using the ClustalW program in Bioedit v. 7.2.5. The sequences of tyrosinases from various organisms and their accession numbers are given inside the brackets: *B. aryabhattai* TFG5 (MW505907), *B. aryabhattai* (WP_043981293.1), *B. megaterium* (3NM8), *Bacillus* sp. Root147 (WP_057234811), *B. flexus* (WP_025750930), *Fictibacillus macauensis* (WP_050979754), *B. macauensis* ZFHKF-1 (EIT84795), *Pseudomonas veronii*(WP_017849537), *Nitrosomonas europaea* ATCC 19718 (CAD85152), *P. fluorescens* (WP_047297073), *Streptomyces tsukubensis* (WP_040914590), *Streptomyces* sp. NRRL S-87 (WP_030192477), *Brevibacillus* sp. Is *Brevibacillus laterosporus* GI-9 (CCF17084), *S. roseus* (WP_048477298), and *S. roseoverticillatus* (WP_030366439). The copper-binding His and Phe sites are boxed. The color shades represent the identical and conserved amino acids in all the organisms. **Table S1.** FTIR reaction products from the phenols and the formation of additional functional groups. **Table S2.** FT-IR functional groups and their corresponding wavenumber of CWW.

## Data Availability

All data of this manuscript are included in the manuscript. No separate external data source is required. Any additional information required will be provided by communicating with the corresponding author via the official mail: usiva@tnau.ac.in.

## Abbreviations

HS: Humic substances
TFG5: Termite fungal Garden isolate 5
CO_2_: Carbon di oxide
Gt: Gigatons
SOC: Soil organic carbon
SIC: Soil inorganic carbon
LiP: Lignin peroxidase
MnP: Mn-dependent peroxidase
VP: Versatile peroxidase
CWW: Coir pith wash water
ORF: Open Reading Frame
TyrB: Extracellular tyrosinase from *Bacillus aryabhattai* TFG5
FT-IR: Fourier Transformation Infrared Spectroscopy
