# Supplementary material for: Bacillus aryabhattai TFG5-mediated synthesis of humic substances from coir pith wastes
Source: Microb Cell Fact. 2021 Feb 17;20:48. doi: 10.1186/s12934-021-01538-x (PMC7891170; doi:10.1186/s12934-021-01538-x)
Supplement: Supplementary file 1 — Additional file 1: Figure. S1. Multiple sequence alignment of TyrB: MSA was performed using the ClustalW program in Bioedit v. 7.2.5. The sequences of tyrosinases from various organisms and their accession numbers are given inside the brackets: B. aryabhattai TFG5 (MW505907), B. aryabhattai (WP_043981293.1), B. megaterium (3NM8), Bacillus sp. Root147 (WP_057234811), B. flexus (WP_025750930), Fictibacillus macauensis (WP_050979754), B. macauensis ZFHKF-1 (EIT84795), Pseudomonas veronii(WP_017849537), Nitrosomonas europaea ATCC 19718 (CAD85152), P. fluorescens (WP_047297073), Streptomyces tsukubensis (WP_040914590), Streptomyces sp. NRRL S-87 (WP_030192477), Brevibacillus sp. Is Brevibacillus laterosporus GI-9 (CCF17084), S. roseus (WP_048477298), and S. roseoverticillatus (WP_030366439). The copper-binding His and Phe sites are boxed. The color shades represent the identical and conserved amino acids in all the organisms. Table S1. FTIR reaction products from the phenols and the formation of additional functional groups. Table S2. FT-IR functional groups and their corresponding wavenumber of CWW. [file 12934_2021_1538_MOESM1_ESM.docx]

**Additional file 1**

**Fig. S1:** MSA was performed using ClustalW program using Bioedit v. 7.2.5. The tyrosinase sequences from the various organisms and their accession numbers are given inside ear bracket are *B. aryabhattai* TFG5 (MW505907), *B. aryabhattai* (WP_043981293.1), *B. megaterium* (3NM8), *Bacillus* sp. Root147 (WP_057234811), *B. flexus* (WP_025750930), *Fictibacillus macauensis* (WP_050979754), *B. macauensis* ZFHKF-1 (EIT84795), *Pseudomonas veronii* (WP_017849537), *Nitrosomonas europaea* ATCC 19718 (CAD85152), *P. fluorescens* (WP_047297073), *Streptomyces tsukubensis* (WP_040914590), *Streptomyces* sp. NRRL S-87 (WP_030192477), *Brevibacillus* sp. is *Brevibacillus laterosporus* GI-9 (CCF17084), *S. roseus* (WP_048477298), *S. roseoverticillatus* (WP_030366439). The copper-binding His sites and Phe site are boxed. The color shades represent identical and conserved amino acids in all the organisms.

**Table S1.** FTIR reaction products from the phenols and the formation of additional functional groups

| **Monomers** | **Wavenumber**  **(cm^-1^)** | **Tyrosinase from *B. aryabhattai* TFG5** | **Wavenumber**  **(cm^-1^)** | **Control (without enzyme)** |
| --- | --- | --- | --- | --- |
| *p*-Hydroxy benzoic acid | 575.647 cm^-1^ | Alkyl halides C-Br-strong-stretching | 575.647 cm^-1^ | Alkyl halides C-Br-strong-stretching |
|  | **1327.5 cm^-1^** | **strong C-N stretch-aromatic amines** |  |  |
|  | 1635.37 cm^-1^ | Alkenes C=C-Variable-Stretching Non conjugated C=C | 1635.37 cm^-1^ | Alkenes C=C-Variable-StretchingNon conjugated C=C |
|  | 2159.98 cm^-1^ | Akynes CΞC Stretch | 2161.81 cm^-1^ | Akynes CΞC Stretch |
|  | 3341.43 cm^-1^ | Alcohol OH-Variable-Stretching hydrogen bonded | 3341.43 cm^-1^ | Alcohol OH-Variable-Stretching hydrogen bonded |
| Catechol | 575.683 cm^-1^ | Alkyl halides C-Br-strong-stretching | 575.683 cm^-1^ | Alkyl halides C-Br-strong-stretching |
|  | 1636.37 cm^-1^ | Alkenes C=C-Variable-Stretching Non conjugated C=C | 1636.37 cm^-1^ | Alkenes C=C-Variable-Stretching Non conjugated C=C |
|  | **2129.98 cm^-1^** | **Akynes CΞC Stretch** |  |  |
|  | 3341.43 cm^-1^ | Alcohol OH-Variable-Stretching hydrogen bonded | 3341.43 cm^-1^ | Alcohol OH-Variable-Stretching hydrogen bonded |
| L- Dopa | 564.07 cm^-1^ | Alkyl halides C-Br-strong-stretching | 530.28 cm^-1^ | Alkyl halides C-Br-strong-stretching |
|  | 1636.3 cm^-1^ | Alkenes C=C-Variable-Stretching Non conjugated C=C | 1635.3cm^-1^ | Alkenes C=C-Variable-Stretching Non conjugated C=C |
|  | 1738.51cm^-1^ | C=O Strong- stretching-saturated aliphatic aldehyde | 1738.51cm^-1^ | C=O Strong- stretching-saturated aliphatic aldehyde |
|  | **2147.35 cm^-1^** | **Akynes CΞC Stretch** |  |  |
|  | 3327.57 cm^-1^ | Alcohol OH-Variable-Stretching hydrogen bonded | 3340.1 cm^-1^ | Alcohol OH-Variable-Stretching hydrogen bonded |
| 2,6 Dimethoxy phenol | 592.039 cm^-1^ | Alkyl halides C-Br-strong-stretching | 588.182 cm^-1^ | Alkyl halides C-Br-strong-stretching |
|  | 1326 cm^-1^ | Alcohol OH Medium- Deformation | 1310.39 cm^-1^ | Alcohol OH Medium- Deformation |
|  | 1636.3 cm^-1^ | Alkenes C=C-Variable-StretchingNon conjugated C=C | 1636.3 cm^-1^ | Alkenes C=C-Variable-StretchingNon conjugated C=C |
|  | 2148.35 cm^-1^ | Akynes CΞC Stretch | 2120.35 cm^-1^ | Akynes CΞC Stretch |
|  | 3340.1 cm^-1^ | Alcohol OH-Variable-Stretching hydrogen bonded | 3340.1 cm^-1^ | Alcohol OH-Variable-Stretching hydrogen bonded |
| *p*-Nitro phenol | 1654.62 cm^-1^ | Alkenes C=C-Variable-StretchingNon conjugated C=C | 1635.34 cm^-1^ | Alkenes C=C-Variable-Stretching Non conjugated C=C |
|  | 2191.7 cm^-1^ | Akynes CΞC Stretch | 2183.99 cm^-1^ | Akynes CΞC Stretch |
|  | 3291.89 cm^-1^ | Alcohol OH-Variable-Stretching hydrogen bonded | 3341.07 cm^-1^ | Alcohol OH-Variable-Stretching hydrogen bonded |
| *p*-Cresol | 540.613 cm^-1^ | Alkyl halides C-Br-strong-stretching |  |  |
|  | 1367.28 cm^-1^ | Alkanes CH rock | 1367.28 cm^-1^ | Alkanes CH rock |
|  | **1655.59 cm^-1^** | **Alkenes C=C Stretching Medium** |  |  |
|  | **2185.92 cm^-1^** | **Alkynes CΞC Stretch** |  |  |
|  | 3451.085cm^-1^ | Alcohol OH-Variable-Stretching hydrogen bonded | 3455.81cm^-1^ | Alcohol OH-Variable-Stretching hydrogen bonded |

**Table S2.** FT-IR functional groups and their corresponding wavenumber of CWW

| Sample | Wave number cm^-1^ | Corresponding functional group |
| --- | --- | --- |
| Unsterilized CWW (Control) | 412.7 | Aromatic ring medium to strong deformation |
|  | 1449 | Aromatic ring medium stretching |
|  | 1492 | Aromatic ring variable stretching |
|  | 1580 | Aromatic ring medium stretching |
|  | 1605 | Aromatic ring variable stretching |
| Filter sterilized CWW | 722.2 | Amines NH strong wagging broad peak |
|  | 1143.4 | Amines C-N stretching medium |
|  | 1608 | Amines NH variable deformation |
|  | 3443 | NH weak stretching |
|  |  |  |
| Sterilized CWW | 696.9 | Aromatic ring strong bending out of plane ring bending |
|  | 747 | Aromatic ring strong deformation 6 adjacent H out of plane deformation |
|  | 878.6 | CH medium deformation 5 adjacent H out of plane deformation |
|  | 991.2 | CH weak bending in plane H bend |
|  | 1066.6 | CH weak bending in plane H bend |
|  | 1390.4 | OH medium deformation |
|  | 1436.4 | Ring medium stretching |
|  | 1492.8 | Ring variable stretching |
|  | 1605 | Ring variable stretching |
|  | 1643 | Alkenes C=C variable non conjugated C=C medium stretching |
|  | 3022 | Alkenes C-H medium stretching |
|  | 3642 | OH medium stretching in sharp peak |
